# Molecular and Cellular Mechanisms of Epilepsy 2.0

**DOI:** 10.3390/ijms242417464

**Published:** 2023-12-14

**Authors:** Aleksey V. Zaitsev

**Affiliations:** Sechenov Institute of Evolutionary Physiology and Biochemistry, 194223 Saint Petersburg, Russia; aleksey_zaitsev@mail.ru

Epilepsy is a prevalent neurological disorder. According to the World Health Organization (WHO), approximately 70 million people worldwide have epilepsy. This disorder is characterized by periodic and spontaneous seizures caused by the hyperexcitability and hypersynchronization of brain neurons [1]. Epilepsy can manifest itself in various forms and have a significant impact on an individual’s quality of life [2]. Epilepsy is a heterogeneous disease that is linked to different etiological factors, such as genetic, infectious, traumatic, vascular, toxic, tumor, etc. Despite the availability of many antiseizure drugs, about 30% of epilepsy patients, especially those with temporal lobe epilepsy (TLE), continue to experience seizures. One reason for this might be that most current antiseizure drugs focus on a single “terminal” mechanism of epilepsy. For example, the main anticonvulsants aim to (1) modulate voltage-gated sodium, calcium or potassium channels; (2) enhance GABA-mediated inhibitory neurotransmission; (3) attenuate glutamate-mediated excitatory neurotransmission; and (4) modulate neurotransmitter release via a presynaptic action [3].

However, the most rational therapeutic option for drug-resistant epilepsy is preventing the development and progression of epilepsy, based on an understanding of the pathophysiological mechanisms of epileptogenesis [4,5]. In recent decades, progress has been made in the field of epilepsy due to advancements in genetics, resulting in a better understanding of the pathophysiological basis for epilepsy syndromes and epileptic encephalopathies [6]. Currently, genome-wide association studies (GWAS) have linked specific low-level somatic mutations with intractable epilepsy, identifying multiple genes at risk of epilepsy (see for review [7,8]).

This Special Issue, “Molecular and Cellular Mechanisms of Epilepsy 2.0”, emphasizes the breakthroughs that have been made in identifying the molecular, cellular, and network alterations associated with severe epilepsy. Shevlyakov et al. [9] used a computational in silico approach to generate a comprehensive network of molecular pathways involved in epilepsy based on known human epilepsy candidate genes and their established molecular interactors. The authors found several other gene clusters beyond neuronal hyperactivity, including genes not directly related to epilepsy but otherwise critical to CNS function and worthy of further investigation. Among such gene clusters, the following deserve a special mention: (1) mitochondrial and metabolic genes; (2) the mammalian target of rapamycin (mTOR) pathway; (3) transcription factors and chromatin remodeling genes; (4) cytoskeleton and cell division. The authors examine the relationships between these different genetic factors and show that abnormalities in one cluster can contribute to dysfunction in many cellular systems. For instance, mutations in the mitochondrial genome can affect other, higher-level systems that may also be relevant to the pathogenesis of epilepsy. Indeed, considering the dependence of many molecular processes on ATP, mutations in mitochondrial genes pose a significant risk of disrupting such ATP-dependent mechanisms.

Metabolic and mitochondrial disorders are known to cause epilepsy, including several common types such as glucose transporter (GLUT1) deficiency syndrome, hypoglycemia, creatine deficits, and mitochondrial encephalomyopathies. A shared characteristic of these widespread metabolic disorders, leading to epileptic seizures, is a reduction in energy compounds that results in decreased ATP levels [10]. These data imply that seizures can be managed by influencing metabolic pathways. The ketogenic diet [11] is a well-known strategy, as are drugs that impede metabolism, such as 2-deoxyglucose [12] and stiripentol [13], which demonstrate antiepileptic effects, indicating the potential of the metabolic management of epilepsy. Epilepsy treatment still requires the identification of more effective therapeutic targets, and the metabolic pathway presents a promising alternative [10].

Mutations in genes within the mTOR pathway are also a common cause of epilepsy [14]. These mutations often occur alongside focal cortical dysplasia (PCD) and other malformations in the cortex [15]. Hyperactivated mTOR plays a critical role in the development of acquired epilepsy in animal models. Various studies have shown that mTOR inhibitors, such as rapamycin and its analogs, can reduce seizure development and prevent epileptogenesis-related mechanisms in many animal models. Additionally, some studies have also demonstrated anticonvulsant activity. Therefore, this pathway has been recognized as a potential target for novel therapeutic strategies in the treatment of epilepsy and epileptogenesis [16].

A promising therapeutic approach to preventing epileptogenesis is to influence peroxisome proliferator-activated receptors (PPARs) [17]. PPARs are nuclear transcription factors that regulate metabolic and tissue development processes. They are involved in various physiological functions and have been linked to metabolic diseases, lipid metabolism, and inflammation control [18,19]. PPARs can be activated by certain ligands, such as short-chain fatty acids, which are metabolites produced by the gut microbiota. An experimental study published in this Special Issue by Zubareva et al. examines the effects of *Bifidobacterium longum*, a probiotic, on inflammation, neuronal degeneration, and behavior in a lithium–pilocarpine model of temporal lobe epilepsy (TLE) induced in young adult rats [20]. The results indicate that *B. longum* may have a beneficial effect on TLE and may provide valuable insights into the role of gut microbiota in epileptogenesis.

When searching for and selecting effective anticonvulsants, it is crucial to consider that pharmacoresistance may result from genetic factors. Brancati et al. analyzed the effects of single-nucleotide polymorphisms (SNPs) in genes involved in the pharmacokinetics and pharmacodynamics of antiseizure drugs and assessed whether these polymorphisms could impact the efficacy, blood levels, and clinical outcomes of treatment in patients with epilepsy. Interindividual variability in drug response can be influenced by SNPs in genes that encode drug efflux transporters (ABCB1, ABCC2) located in the gastrointestinal tract and blood–brain barrier, brain targets of antiseizure drugs (voltage-gated sodium channels, synaptic vesicle protein SV2A), and enzymes involved in drug metabolism (CYP2C19, UGT1A4) [21]. The authors conclude that SNPs in enzymes and transporters have an impact on the pharmacokinetics of anticonvulsants. Therefore, population pharmacokinetic modeling that incorporates the genotypes of drug-metabolizing enzymes and transporters can be a highly effective tool in determining personalized dosing regimens for antiseizure therapy. Clinicians can rely on genetic and non-genetic factors affecting enzyme activity in choosing the most suitable anticonvulsants and adjusting dosage levels for their patients.

In conclusion, this Special Issue highlights a range of techniques used to study the molecular and cellular mechanisms of epilepsy. Advancing our understanding of the underlying molecular mechanisms of epilepsy and epileptogenesis, along with incorporating innovative drug, cell, and gene therapy approaches into carefully controlled clinical trials, will promote significant progress in a field with many unmet needs.
